# Microbial Community Variations and Bioconversion Improvements during Soybean-Based Fermentation by Kefir Grains

**DOI:** 10.3390/foods12081588

**Published:** 2023-04-08

**Authors:** Jiaqi Luo, Siyu Liu, Hongyun Lu, Qihe Chen, Ying Shi

**Affiliations:** 1 Department of Food Science and Nutrition, Zhejiang University, Hangzhou 310058, China; 2 Future Food Laboratory, Innovation Center of Yangtze River Delta, Zhejiang University, Jiaxing 314100, China

**Keywords:** soybean, kefir grains, third-generation sequencing, fermentation, isoflavones

## Abstract

Soybeans possess unexpected flavors and are difficult to be absorbed by the gastrointestinal tract. Kefir grain fermentation provides diverse strains and bioactive compounds, which may enhance flavor and bioaccessibility. Third-generation sequencing was applied to analyze the microbial diversity in milk and soybean kefir grains in this study. In both types of kefir grains, the most common bacterial genus was *Lactobacillus*, and their fungal communities were dominated by *Kazachstania*. *Lactobacillus kefiranofaciens* was the most abundant species in kefir grains, while *Lactobacillus kefiri* showed a higher proportion in soybean kefir grains. In addition, the quantification of free amino acids and volatile flavor compounds in soybean solution and soybean kefir have shown the increased content of glutamic acid and a decreased amount of unpleasant beany flavor compounds, demonstrating that the nutritive value and sensory properties of soybean can be improved by kefir grain fermentation. Finally, the bioconversion of isoflavones during fermentation and in vitro digestion was evaluated, suggesting that fermentation is beneficial for aglycone formation and absorption. To conclude, kefir fermentation is proposed to change the microbial structure of kefir grains, promote the nutritional value of soybean-based fermented products, and provide possible solutions for the development of soybean products.

## 1. Introduction

Kefir grain is a multi-strain symbiotic system composed of a mixture of varieties of microbial species, proteins, and exopolysaccharides [1]. It generally acts as a starter for a fermented dairy product, called kefir, which provides diverse bioactive compounds including amino acids, peptides, and polysaccharides, such as kefiran [2,3]. Moreover, milk kefir is conferred plenty of bioactive properties during fermentation, such as antioxidant, antiallergic, and anti-inflammatory effects, etc., thus providing benefits to gut microbiota and human health [2,4,5]. However, more and more novel plant-based food products have been studied and developed innovatively due to the increasing demand for healthy food. Especially for consumers who are sensitive and intolerant to lactose or allergic to casein, some soybean-sourced foods are developed and consumed extensively [6]. Soybean products and soybean derivatives have been a major focus of existing research because they possess valuable nutrients, including proteins, isoflavones, phenolic compounds, and other compounds that are beneficial for human health, for example, by lowering the risks of hypertension, cancer, cardiovascular diseases, and osteoporosis [7,8,9]. In contrast, the bioaccessibility of isoflavones in soybean foods is relatively low because isoflavones generally exist as glucoside forms. As reported, glucosides can be transformed into aglycone forms by fermenting [10,11], and aglycone exhibits a higher bioavailability than glucoside. Therefore, promoting the biotransformation of glucoside to aglycone can improve the bioactivity of soybean foods. Meanwhile, some studies have suggested that many bacteria are helpful for isoflavone glucoside hydrolyzation and the biological activity improvement of soybean foods [9]. The fermented soymilk obtained by lactic acid bacteria fermentation has been studied and developed as a novel plant-based drink with a smooth texture and reduced beany flavor [12]. Meanwhile, its nutritional characteristics and health-beneficial properties have been developed for application [12,13].

Presently, the microbial diversity, including both bacteria and fungi of kefir grains, and bioactivities of milk kefir have been well studied [14]. In addition, some nutritional components and bioactivities of non-milk kefir have been investigated as well [7]. It is known that different microbial species, including bacteria and fungi in kefir grains, can mutually influence the chemical composition of the kefir products. Microbial diversity is also affected by different culture environments [2,15]. In contrast, the altered microbial structure of kefir grains growing in a plant protein is relatively less investigated and reported. In this study, novel soybean kefir grains and soybean kefir were cultured and prepared accordingly. Moreover, the profile of microbial diversity involving the species and abundance of lactic bacteria and yeasts was investigated using third-generation sequencing technology (also named single-molecule sequencing technology). In addition, the concentration of free amino acids and volatile compounds in soybean solution and soybean kefir was assessed. Furthermore, in vitro gastrointestinal digestion simulation was ultimately applied to evaluate the bioconversion and bioavailability of isoflavones in soybean kefir.

## 2. Materials and Methods

### 2.1. Chemicals and Reagents

Kefir grains from Hebei, China, were used in this study. Soybeans and milk were purchased in the supermarket. Pepsin was obtained from Macklin Inc (Shanghai, China), and pancreatin was provided by Yuanye Biotechnology Co., Ltd. (Shanghai, China). Methanol and acetonitrile were HPLC-grade and obtained from Aladdin Biotechnology Co., Ltd. (Shanghai, China). All other chemicals and reagents used were of analytical grade.

### 2.2. Sampling and Community Enrichment

Kefir grains from Hebei, China, were used in this study. The grains were divided into two groups: one group was cultured with milk for 30 days and labeled kefir, and the other group was cultured with soybean solution for 30 days and labeled soy. The kefir grains were filtered out with a sterile strainer and ground with the tissuelyser (Tissuelyser-48, Jingxin tech, Shanghai). Four replicate kefir grain samples were taken from broken and homogenized kefir grains, and three replicate soybean kefir grain samples were taken from soybean kefir grains randomly.

### 2.3. Extraction of DNA and Sequencing

The extraction of DNA from samples was conducted using the FastDNA spin kit for soil (MP Biomedicals, CA, USA) according to the instructions of the manufacturer. After genomic DNA extraction was completed, the extracted genomic DNA was detected by 1% agarose gel electrophoresis. Specific primers with barcodes were synthesized for PCR amplification according to the specified sequencing region. TransStart Fastpfu DNA polymerase was used for PCR (9700, ABI, USA). Primers 27F (5′-AGRGTTYGATYMTGGCTCAG-3′) and 1492R (5′-RGYTACCTTGTTACGACTT-3′) were used to amplify bacterial 16 S rDNA, and ITS1F (5′-CTTGGTCATTTAGAGGAAGTAA-3′) and ITS4R (5′-TCCTCCGCTTATTGATATGC-3′) were used to amplify the fungal 18S rDNA-ITS sequence. The specific conditions of PCR amplification were set as described [16]. The quality of PCR products was determined by 2% agarose gel electrophoresis after amplification.

### 2.4. Genome Sequencing and Bioinformatics Analysis

The sequencing process was performed on a PacBio-based SMRT sequencing platform (Pacific Bioscience, Menlo Park, CA, USA) from MajorBio Bio-pharm Technology Co, Ltd. (Shanghai, China). The circular consensus sequencing (CSS) algorithm was used for raw reads processing, and then quality control and filtering were conducted to assess sequence quality. Usearch software (version 11) was applied for operational taxonomic units clustering. OTU cluster analysis and species taxonomic analysis were performed after distinguishing samples (Uparse, version 11). Subsequent statistical and visual analyses were carried out on the MajorBio Cloud platform (http://cloud.majorbio.com/ (accessed on 18 August 2022).

A total of 204,741 bacterial and 455,547 fungal sequences were obtained from samples by SMRT sequencing. To eliminate the effects of differences in the number of optimized sequences produced by each sample, the data volume of all samples was randomly extracted to the same data volume according to the fewest sequences for a subsequent analysis. The operational taxonomic unit (OTU) annotation suggested that a total of 2 domains, 2 kingdoms, 14 phyla, 20 classes, 46 orders, 66 families, 93 genera, 162 species, and 3875 OTUs were measured in bacteria. A total of 1 domain, 1 kingdom, 2 phyla, 6 classes, 9 orders, 14 families, 17 genera, 21 species, and 60 OTUs were measured in fungi.

### 2.5. Preparation of Soybean Kefir

Soybeans (100 g) were soaked in 1000 mL of pure water for 3–4 h at room temperature (20 °C). Afterwards, the soaked soybeans were ground using a commercial blender and boiled for about 5–10 min, which was followed by filtration, cooling, and storage at 4 °C to prepare the soybean solution for further use. Subsequently, the soybean solution was inoculated with kefir grains at 2% (*w*/*v*) and fermented at 28 °C for 36–48 h to prepare the soybean kefir.

### 2.6. Analysis of Free Amino Acids and Volatile Flavor Compounds in the Soybean Solution and Soybean Kefir

The amino acid compositions of soybean solution and soybean kefir were evaluated using an automatic amino acid analyzer (L-8900, Hitachi, Japan). Volatile compounds were isolated and identified by SPEM-GC/MS (5973, Agilent Technologies, Palo Alto, CA, USA) according to Rutkowska et al. [17] with some modifications. Subsequently, 5 mL samples were placed into glass vials, equilibrated for 30 min at 35 °C, and stirred using a magnetic stirrer for 30 min. The volatiles were extracted into the needle and transferred to the column in GC/MS. The mass spectra were compared to the NIST library for compound identification, and their relative abundances were calculated according to the corresponding peak areas.

### 2.7. In Vitro Digestion Process of Soybean Solution and Soybean Kefir

In this study, the simulated in vitro gastrointestinal digestion involves the gastric phase and intestinal phase and was performed according to the methods of Simsek et al. [18] and Aytekin et al. [19] with some modifications. A total of 15 mL of soybean solution/soybean kefir was mixed with an equal volume of simulated gastric fluids and 0.3 M of CaCl_2_. Then, pepsin (>3000 U/mg) was added (0.2%, *w*/*v*). The pH of the mixture was adjusted to 2.0, and the solution was incubated at 37 °C for 2 h with shaking at 200 rpm. After gastric digestion, the chime was transferred to an equal volume of simulated intestinal fluids for the subsequent intestinal phase. The pH was adjusted to 6.8 with 1 M of NaOH, and the mixture was incubated at 37 °C for 2 h with shaking at 200 rpm. After stopping the reaction, the digested soybean solution/soybean kefir were prepared and stored at −20 °C for later use.

### 2.8. Extraction, Separation, and Quantification of Isoflavones from Soybean Solution and Soybean Kefir

We determined isoflavones using HPLC based on the methods described by Ningtyas et al. [10] and Salces et al. [20]. The lyophilized samples of soybean solution and soybean kefir were defatted with hexane (1:10, *w*/*v*) in an ultrasound bath for 20 min. Next, 80% methanol was added for isoflavone extraction in an ultrasound bath for 15 min. The mixtures were subsequently centrifuged at 8000× *g* for 10 min, which was followed by separating the interlayer phase and filtrating using a filter syringe (0.22 μm) for HPLC detection. Isoflavone analyses were conducted using a Waters HPLC system (e 2675, Waters, Beijing, China) equipped with an HPLC pump, an autosampler, a vacuum degasser, a UV detector (260 nm), a thermostatically controlled column compartment, and a 5C18-MS-II packed column (COSMOSIL, Nacalai Tesque Inc., Kyoto, Japan). The temperature of the column was set at 25 °C with a 5 μL injection volume. The mobile phases include solvent A (0.1% phosphoric acid in water) and solvent B (0.1% phosphoric acid in 80% acetonitrile), and the flow rate was 1 mL/min.

### 2.9. Statistical Analysis

The experiments were implemented in triplicate independently, and the results were represented as mean ± standard deviation. A one-way analysis of variance (ANOVA) and *t*-test were used to analyze the differences between groups. These data were processed and analyzed using Graphpad Prism 7.00 (GraphPad Software, Inc., San Diego, CA, USA).

## 3. Results

### 3.1. Sequencing Sample Size, Depth, and Alpha Diversity

**Figure 1:**
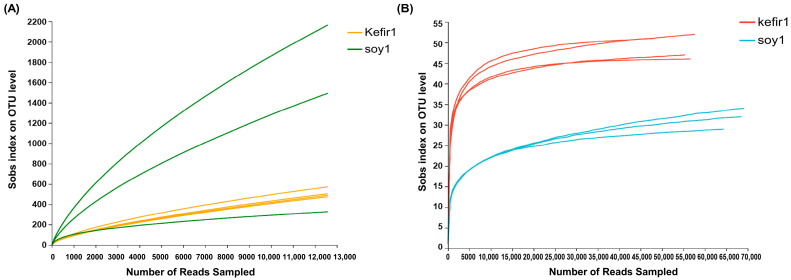
Rarefaction curves of bacterial (**A**) and fungal (**B**) communities in kefir grains and soybean kefir grains.

The number of sequences and their corresponding OTUs was used to construct the rarefaction curves by random sampling [21]. As suggested by the alpha rarefaction plots (Figure 1), curves showed that the reads were sufficient to cover the entire population and can well represent the microbial community, including bacteria and fungi. The majority of microbial types present in kefir grains and soybean kefir grains had already been captured, and the alpha rarefaction curves illustrated that the sequencing depth was sufficient to reflect the diversity in the samples [22]. Moreover, the bacterial diversity of the kefir grains was lower than that of the soybean kefir grains, while the fungal diversity of the kefir grains was higher than that of the soybean kefir grains.

**Figure 2:**
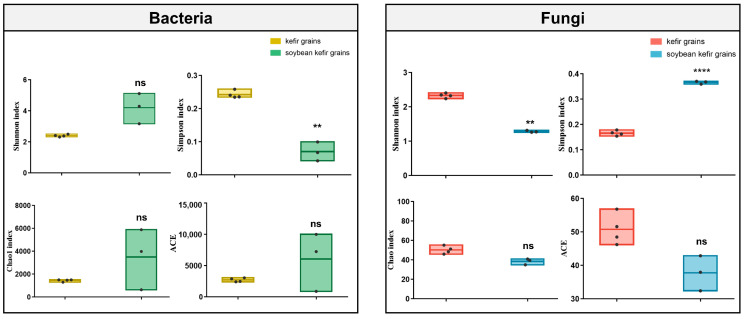
Alpha diversity indices in kefir grains and soybean kefir grains. The significance was denoted by asterisks. ** *p* < 0.01, **** *p* < 0.0001, and ns indicates no significance.

Alpha diversity analysis shows the diversity and richness of the bacterial and fungal community in a sample [22]. Four statistical analysis indices, including Shannon, Simpson, Chao, and ACE (abundance-based coverage estimator), were used in this research to investigate diversity and richness. The alpha diversity indices in the samples are listed in Figure 2. The ACE and Chao 1 indices showed the higher bacterial species richness of the soybean kefir grains group and the higher fungal species richness of the kefir grains group. Moreover, the Simpson diversity indices indicated that the kefir grains and soybean kefir grains possess higher bacterial and fungal taxonomic diversity, respectively. The significant difference in Simpson indices between groups suggested the noteworthy effects on the microbial diversity of kefir grains cultured by the soybean solution.

### 3.2. Discrimination of Similarity in Community Structure with Principal Coordinate Analysis (PCoA)

**Figure 3:**
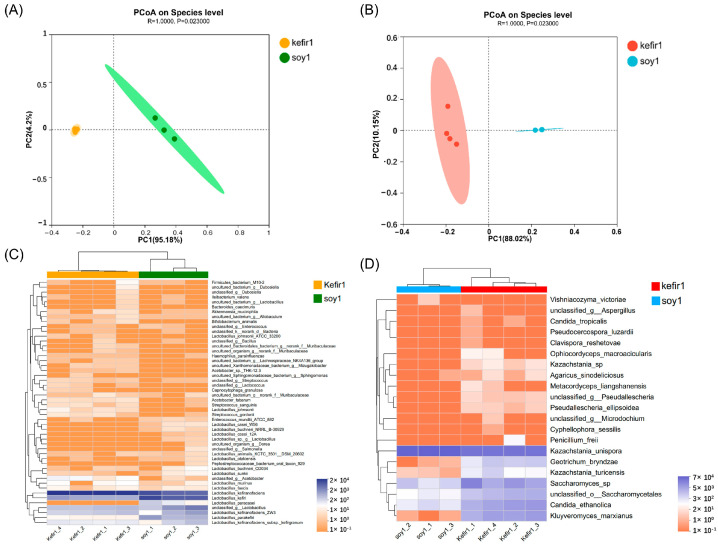
PCoA graphs of bacterial (**A**) and fungal (**B**) communities based on Bray–Curtis distance at the species level. Each point represents an individual sample. Heat map of the relative abundance of predominant bacterial and fungal species in kefir grains and soybean kefir grains ((**C**)—bacteria, (**D**)—fungi).

Community diversity in a single sample can be analyzed by alpha diversity. The third-generation SMRT sequencing technique presently exhibits a higher resolution than second-generation HTS sequencing due to the generation of longer reads. However, the fungal annotation databases still need to be improved and expanded at the species level [16]. PCoA based on the Bray–Curtis distance at the species levels was conducted to analyze the correlations between community structures of bacteria and fungi in kefir grain and soybean kefir grain samples, and the graphs are shown in Figure 3A,B, respectively.

As shown in Figure 3A,B, the principal coordinates 1 and 2 (PC1 and PC2) of bacteria occupied 95.18% and 4.2%, with a sum of 99.38%, and PC1 and PC2 of fungi occupied 88.02% and 10.15%, with a sum of 98.17%. The total of PC1 and PC2 of both bacteria and fungi exceeded 50%, implying that the two principal axes explain the variations in the microbial compositions of the samples. Thus, the two main axes can be the basis of the subsequent analysis [16,23].

Samples in the two groups were well clustered in their respective confidence ellipses, suggesting a relatively similar bacterial and fungal species composition in the samples. Notably, the two groups were clearly separated from each other in the bacterial and fungal analysis, indicating significant differences in the community structures of kefir grains and soybean kefir grains.

**Figure 4:**
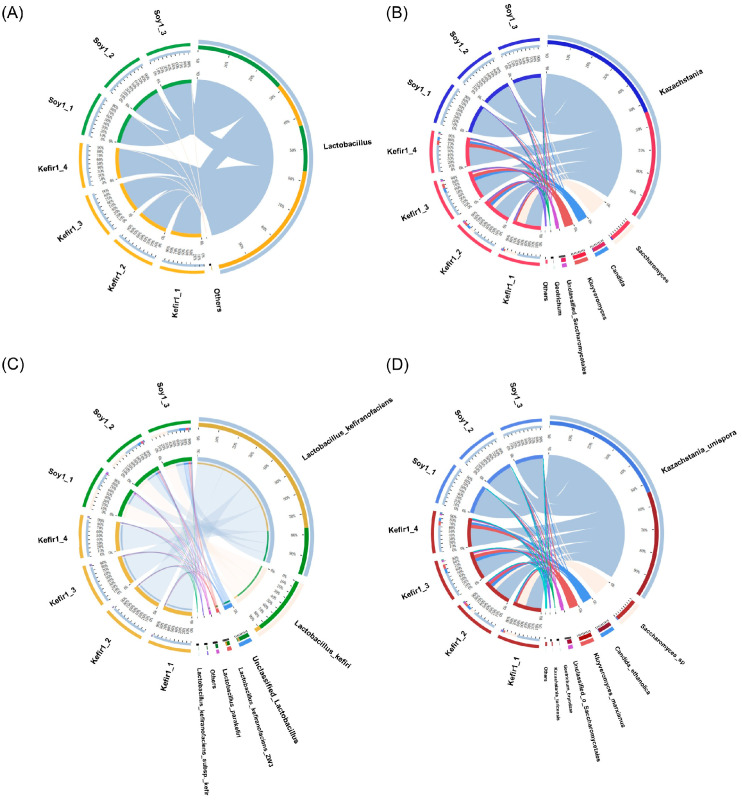
Circos diagram of bacterial (**A**) and fungal (**B**) communities at the genus level, and bacterial (**C**) and fungal (**D**) communities at the species level.

As shown in Figure 4, Circos diagrams at the genus and species level were created for a further visualization of the dominant species proportions in the different sample groups [16,24]. As shown, *Lactobacillus* was the most abundant bacterial genus in both kefir and soybean kefir grains. The major fungal genus was *Kazachstania* in both groups. Meanwhile, *Saccharomyces*, *Candida*, and *Kluyveromyces* were also the dominant genera of the fungal community in kefir grains. At the species level, *Lactobacillus kefiranofaciens* and *Lactobacillus kefiri* comprised the top two species in both groups. In particular, the abundance of *Lactobacillus kefiranofaciens* in kefir grains was higher, while the proportion of *Lactobacillus kefiri* in soybean kefir grains was larger. Additionally, *Kazachstania unispora* dominated the fungal community in both groups.

### 3.3. Analysis of Bacterial and Fungal Community Structure

**Figure 5:**
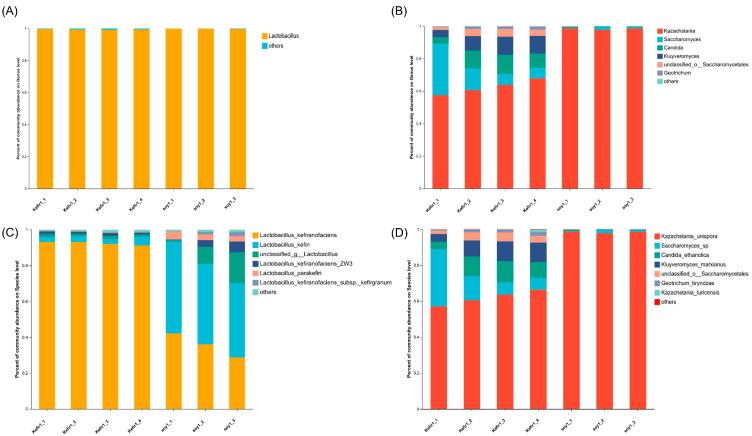
Relative abundance at the genus ((**A**)—bacteria, (**B**)—fungi) and species ((**C**)—bacteria, (**D**)—fungi) levels in kefir grains and soybean kefir grains.

The community composition analysis could intuitively show the community structures of the bacteria and fungi and provide the basis for the subsequent analysis of microbial community differences, associations, evolution, as well as function prediction [16]. Bar diagrams showing the percentages of bacterial and fungal community abundance on genus and species levels are presented in Figure 5.

The most abundant genus in both kefir grains and soybean kefir grains was *Lactobacillus*, which contributed to more than 99% of the total bacteria. *Kazachstania* was the highest relative abundant genus in the fungal community from both kefir and soybean kefir grains, especially in soybean ones (>99%). Moreover, *Saccharomyces*, *Candida*, and *Kluyveromyces* were also the major genera of the fungal community in kefir grains. At the species level of bacteria, the most abundant strain was *Lactobacillus kefiranofaciens* (approximately 93%) in kefir grains, followed by *Lactobacillus kefiri* (about 0.05%). Conversely, the soybean kefir grains group was dominated by *Lactobacillus kefiri* (40~50%), followed by *Lactobacillus kefiranofaciens* (30~40%), *Lactobacillus kefiranofaciens* ZW3 (0.03~0.06%), *Lactobacillus parakefiri* (about 0.04%), and *Lactobacillus kefiranofaciens* subsp. *kefirgranum* (about 0.02%). The fungal species in kefir grains were mainly composed of *Kazachstania unispora* (57~66%), *Saccharomyces* sp. (0.07~0.31%), *Candida ethanolica* (0.04~0.12%), *Kluyveromyces marxianus* (0.04~0.11%), and *Saccharomycetales* (0.02~0.05%). Moreover, the dominant fungal species in soybean kefir grains was *Kazachstania unispora* (>97%). Clustering heat map analysis was performed for a further exploration of the differences in the bacterial and fungal community structures of the two groups (Figure 3C,B) [25,26]. The results reflected that the dominant strains and community structures of bacteria and fungi in kefir grains obviously varied depending on the growing culture.

### 3.4. Analysis of Different Bacterial and Fungal Species in Samples

**Figure 6:**
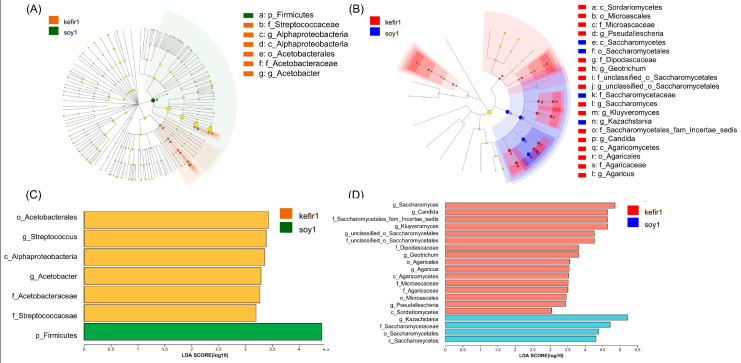
Taxonomic cladograms of bacteria (**A**) and fungi (**B**) produced from LEfSe analysis. Significant discriminant taxon nodes in kefir grains and soybean kefir grains are represented by dark yellow and green for bacteria and red and blue for fungi, and the nodes in light yellow show the OTUs with no significant difference. Bar charts of LDA values of differential bacteria (**C**) and fungi (**D**) in kefir grains and soybean kefir grains (LDA value > 2, *p* < 0.05). The LDA score implies the level of differentiation between the two groups.

Linear discriminant analysis effect size (LEfSe) and linear discriminant analysis (LDA) were conducted to further investigate and evaluate the differences in the structure of the microbiota in kefir grains and soybean kefir grains (Figure 6) [27]. The nodes represented in different colors reflect the OTUs with significant differences, and the sizes of the nodes correspond to the relative abundance of OTUs [28,29]. In the kefir grains, 6 dominant bacterial and 16 dominant fungal taxa were found, as opposed to 1 bacterial and 4 fungal taxa in the soybean kefir grains. The bacteria, including *Streptococcaceae*, *Streptococcus*, *Alphaproteobacteria*, *Acetobacterales*, *Acetobaceraceae*, and *Acetobacter*, were dominant in kefir grains. In contrast, *Firmicutes* were the main phylum in soybean kefir grains. Meanwhile, fungi including *Sordariomycetes*, *Microascales*, *Microascaceae*, *Pseudallescheria*, *Dipodascaceae*, *Geotrichum*, *Saccharomyces*, *Kluyveromyces*, *Candida*, *Agricomycetes*, *Agricales*, *Agricaceae*, and *Agricus* were enriched in the kefir grains. *Saccharomycetes, Saccharomycetales, Saccharomycetaceae*, and *Kazachstania* were the dominant family or order in the soybean kefir grains. The results showed a remarkable variation in the composition and structure of microbiota in kefir grains after culturing in soybean solution, and significant differences between the kefir grains and soybean kefir grains.

### 3.5. Analysis of Free Amino Acid Composition in Soybean Solution and Soybean Kefir

**Figure 7:**
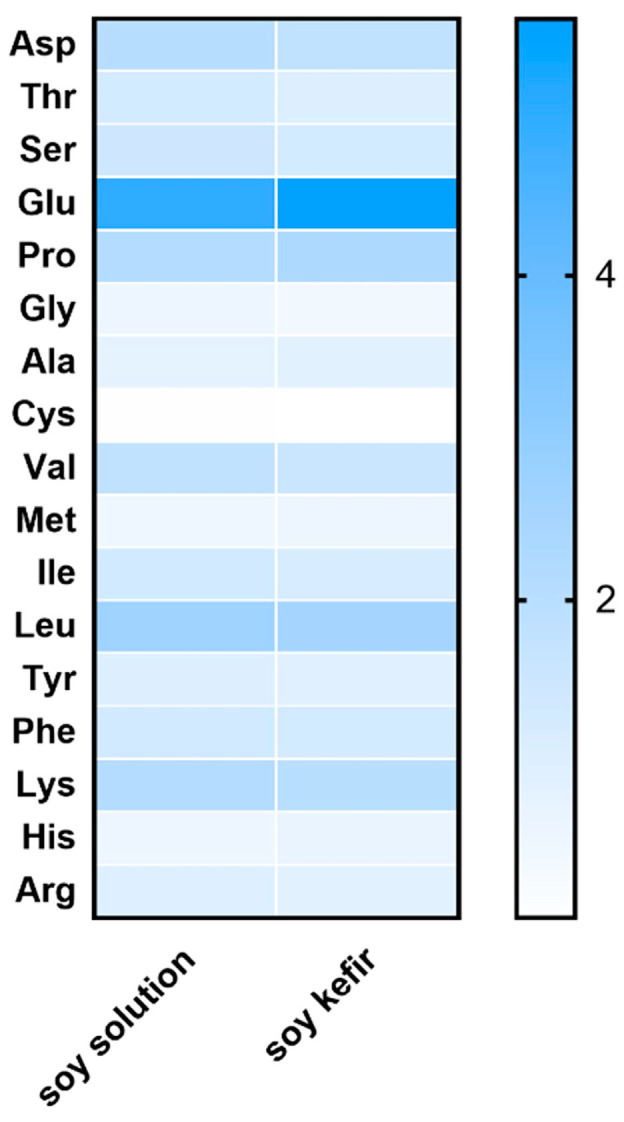
Amino acid profiles of the soybean solution and soybean kefir.

The results of the free amino acid measurement are illustrated in Figure 7. The total amino acid concentration of the soybean solution was 25.49 mg/g, while that of soybean kefir was 24.63 mg/g. The total content of free amino acids in soybean kefir was slightly lower than that in soybean solution, which may be due to the consumption of free amino acids as nutrients by Lactobacillus grown in kefir grains [30,31]. In the process of producing soybean kefir, amino acids can also be produced by yeast metabolism or protein hydrolysis [32]. The highest content of any amino acid in both soybean solution (4.98 mg/g) and soybean kefir (5.58 mg/g) was glutamic acid (Glu). The results showed that the content of Glu increased significantly after kefir fermentation. Leucine (Leu) and proline (Pro) were also detected as major free amino acids in soybean kefir. Similar to the research results of Gamba et al. [6], the contents of free amino acids such as Leu, arginine (Arg), and valine (Val) decreased slightly in the kefir fermentation process of the soybean solution.

### 3.6. The Volatile Flavor Profile in Soybean Solution and Soybean Kefir

**Figure 8:**
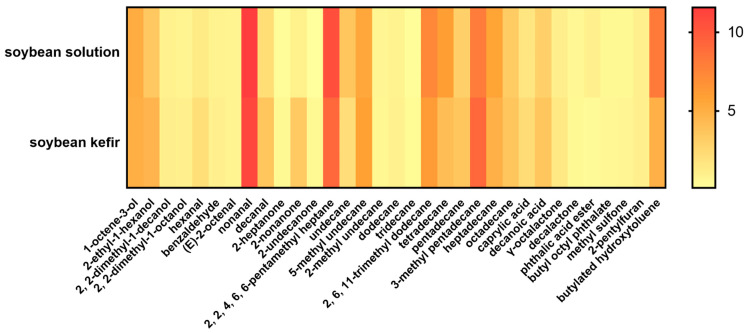
The volatile flavor contents of soybean solution and soybean kefir.

As reported, soybean food was influenced by its unique beany flavor and a slightly spoiled odor in originally fermented soybean products [12,33]. The beany odors generally consist of hexanal, 1-octene-3-ol, benzaldehyde, nonal, etc., and could limit the application of soybeans in food products [33,34]. The volatile substances in the soybean solution and soybean kefir were quantified by GC-MS and shown in Figure 8. A total of 33 volatile compounds were identified, including alcohol, aldehyde, ketone, alkane, acids, esters, furan, and phenols. Similarly, hexanal, benzaldehyde, heptanone, nonanone, 1-octene-3-ol, and 2-pentylfuran were the main volatile compounds in soybean kefir, which contributed to the overall aroma of fermented soybean soymilk [12,35]. The contents of the main contributors of beany flavor compounds, including 1-octene-3-ol, 2-pentylfuran, and nonanol, decreased after kefir fermentation. Therefore, fermentation has been demonstrated to decrease the contents of beany flavor substances, as concluded in previous research [36]. In addition, the existence of beany odors was probably due to the binding of some volatile compounds such as hexanal with the hydrophobic regions of proteins [37]. Moreover, the production of some unpleasant flavor chemicals, such as volatile sulfur substances, was related to the metabolism of amino acids during the fermentation process [12]. Methionine and other sulfur-containing amino acids could serve as the substrate for microbial metabolism and become the source of sulfur-containing flavor compounds. Furthermore, the improved content of acids during fermentation could offer a fresh pleasant flavor for fermented soybean products [33,38].

### 3.7. Bioconversion and Bioaccessibility of Soybean Isoflavones after Kefir Fermentation and In Vitro Digestion

As reported, the isoflavones in soybean and soybean foods were demonstrated to be a mixture of glucoside conjugates. The most abundant glucosides are daidzin and genistin, which can be converted into their corresponding aglycones, daidzein and genistein. In addition, glucosides can be hydrolyzed by bacterial *β*-glucosidase and converted into aglycones [9,10,11]. Isoflavones, including daidzin, genistin, daidzein, and genistein, were separated and identified by HPLC from the soybean solution, soybean kefir, digested soybean solution, and digested soybean kefir. As shown in Table 1, glucoside was the most abundant form of isoflavones in the soybean solution and digested soybean solution, while aglycones were abundant in soybean kefir and digested soybean kefir. The concentrations of daidzin and genistin significantly decreased after kefir fermentation, and the concentrations of daidzein and genistein notably increased. Moreover, the total aglycones in soybean kefir showed a significantly higher concentration than in unfermented soybean solution before and after digestion. Therefore, the isoflavones in glucoside form were bioconverted into their corresponding aglycone form by kefir fermentation, and a similar trend can be observed in digested soybeans and digested soybean kefir [10]. As concluded by Peng et al. [33] and Du et al. [38], these results indicate that fermentation can promote the conversion of glycosides to aglycones. Since the isoflavone glycosides failed to pass through the membrane by passive diffusion but aglycones were absorbed faster, fermented soybean kefir with a predominant content of aglycones demonstrated a higher bioavailability [38,39]. Potentially, varieties of probiotic microbes can offer abundant β-glucosidases for isoflavone bioconversion improvement and aglycone enrichment [9]. Through digestion, the concentration of glucosides in the unfermented soybean solution dropped significantly and was nearly unchanged in the kefir groups. In general, fermentation by kefir grains is beneficial for biological isoflavone bioconversion and bioutilization, which can optimize the profile of isoflavones in soybean foods and enhance the potential of soybean products as functional food ingredients [11].

## 4. Conclusions

In this study, kefir grains growing in soybean solution and fermented soybean kefir were obtained. The bacterial and fungal communities of kefir grains and soybean kefir grains and the differential species were analyzed using third-generation sequencing. Additionally, the measurement of amino acids and flavor components proved that kefir fermentation facilitated the production of nutrients and the formation of pleasant volatile compounds in soybean solution. Furthermore, the bioconversion of isoflavones was also improved, promoting aglycone formation by fermentation and in vitro digestion. The absorption potential of isoflavones in soybean products has been enhanced, speculatively, and needs to be further investigated. In conclusion, the microbial structure of kefir grains showed significant differences in different growing environments. Moreover, kefir fermentation is expected to improve the nutritional value of soybean-based fermented products as functional foods in the future. However, further studies are required for the investigation of other chemical alterations during fermentation and the sustainable growth mechanisms of kefir grains in the plant-based environment.

## Figures and Tables

**Table 1:** Concentrations of isoflavones in soybean solution, soybean kefir, digested soybean solution, and digested soybean kefir.

Sample	Concentration of Isoflavones (mg/g)			
	Daidzin	Genistin	Daidzein	Genistein
Soybean solution	1.35 ± 0.02	1.74 ± 0.02	0.12 ± 0.01	0.05 ± 0.01
Soybean kefir	0.11 ± 0.01****	0.13 ± 0.01 ****	0.74 ± 0.02 ****	1.14 ± 0.01 ****
Digested soybean solution	0.67 ± 0.10	0.81 ± 0.03	0.09 ± 0.01	0.15 ± 0.02
Digested soybean kefir	0.13 ± 0.01****	0.13 ± 0.01 ****	0.77 ± 0.06 ****	0.59 ± 0.02 ****

The significance values for soybean kefir and digested soybean kefir groups in each column were obtained by comparing to the soybean solution and digested soybean solution groups, respectively. They were denoted by asterisks. **** *p* < 0.0001.

## Data Availability

Data will be made available on request.
